# Bragg coherent diffraction imaging with the CITIUS charge-integrating detector

**DOI:** 10.1107/S1600576723004314

**Published:** 2023-06-12

**Authors:** Michael Grimes, Kristof Pauwels, Tobias U. Schülli, Thierry Martin, Pablo Fajardo, Paul-Antoine Douissard, Menyhert Kocsis, Haruki Nishino, Kyosuke Ozaki, Yoshiaki Honjo, Toshiyuki Nishiyama Hiraki, Yasumasa Joti, Takaki Hatsui, Mor Levi, Eugen Rabkin, Steven J. Leake, Marie-Ingrid Richard

**Affiliations:** a Université Grenoble Alpes, CEA Grenoble, IRIG, MEM, NRS, 17 rue des Martyrs, F-38000 Grenoble, France; b ESRF – The European Synchrotron, 71 avenue des Martyrs, F-38000 Grenoble, France; c RIKEN SPring-8 Center, RIKEN, 1-1-1 Kouto, Sayo-cho, Sayo-gun, Hyogo 679-5148, Japan; d Japan Synchrotron Radiation Research Institute, 1-1-1 Kouto, Sayo-cho, Sayo-gun, Hyogo 679-5198, Japan; e Department of Materials Science and Engineering, Technion – Israel Institute of Technology, Haifa, Israel; SLAC National Accelerator Laboratory, Menlo Park, USA

**Keywords:** charge-integrating detectors, Bragg coherent diffraction imaging, fourth-generation synchrotrons, photon-counting detectors, dynamic range

## Abstract

The CITIUS detector is a next-generation high-speed X-ray imaging detector. It has integrating-type pixels and is designed to show a consistent linear response at a frame rate of 17.4 kHz, which results in a saturation count rate of over 30 Mcps pixel^−1^ when operating at an acquisition duty cycle close to 100%, and up to 20 times higher with special extended acquisition modes. Here, its application for Bragg coherent diffraction imaging is demonstrated by taking advantage of the fourth-generation Extremely Brilliant Source of the European Synchrotron (ESRF-EBS, Grenoble, France). The CITIUS detector outperformed a photon-counting detector, similar spatial resolution being achieved (20 ± 6 nm versus 22 ± 9 nm) with greatly reduced acquisition times (23 s versus 200 s). It is also shown how the CITIUS detector can be expected to perform during dynamic Bragg coherent diffraction imaging measurements. Finally, the current limitations of the CITIUS detector and further optimizations for coherent imaging techniques are discussed.

## Abstract 2

Three-dimensional Bragg coherent diffraction imaging with the CITIUS charge-integrating detector is demonstrated.

## 1. Introduction

The advent of photon-counting pixel detectors revolutionized the way that X-ray experiments were executed at large-scale experimental facilities (Ponchut *et al.*, 2004 ▸; Kraft *et al.*, 2009 ▸). They operate on the principle that an absorbed X-ray photon creates an excess of charge carriers within a semiconductor. Under an applied electric field, these charges drift and induce an electrical pulse that can be counted by an application-specific integrated circuit (ASIC) if it exceeds some user-defined threshold level. This threshold criterion effectively results in a ‘noise-free’ detector, yielding an excellent signal-to-noise ratio. As synchrotron sources have become brighter, the scattered photon flux is so intense that the electronics cannot discriminate all the individual photons due to pulse pile-up and, as a result, the detector output saturates (the maximum count rate being a bottleneck, typically below 5–10 Mcps pixel^−1^).

In conventional photon-counting pixel detectors, the in-pixel analogue circuitry limits the maximum count rate. Further issues include the tendency for photon-counting detectors to under-count events where photons impinge directly on pixel borders, known as charge sharing (Chmeissani & Mikulec, 2001 ▸). A reasonable solution is to measure the deposited charge instead, using what is termed a charge-integrating detector. With such technology, multiple X-ray photons impinging simultaneously generate a signal scaling linearly, and the saturation effect inherent to photon-counting detectors is not observed. Additionally, when the system noise of the charge-integrating detector is sufficiently low, it is possible to apply a threshold to discriminate the signal from the noise. In this case, charge-integrating detectors have the potential to attain very high dynamic ranges.

Following the development of fourth-generation synchrotrons (*e.g.* MAX IV in Sweden, ESRF-EBS in France, SPring8-II in Japan, APS-U in the USA or Petra IV in Germany), the scientific need for (i) a high count rate over 20 Mphotons pixel^−1^ s^−1^, (ii) extreme X-ray radiation hardness and (iii) faster recording with good signal-to-noise ratio per frame led to the development of such charge-integrating detectors. Such requirements were further challenged by the advent of X-ray free-electron lasers, and one of the development programmes resulted in the JUNGFRAU detector (Mozzanica *et al.*, 2018 ▸; Leonarski *et al.*, 2018 ▸) which operates at 2 kHz. Another line of work was initiated in 2015 as a collaboration led by RIKEN to develop the CITIUS detector to meet the very demanding needs foreseen for the SPring-8-II upgraded synchrotron. Details of the sensor structure and its performance are reported elsewhere (Hatsui *et al.*, 2023 ▸). Briefly, it has integrating-type pixels (Hatsui & Graafsma, 2015 ▸) with a pixel size of 72.6 µm. In standard mode, CITIUS is designed to operate with an equivalent saturation count rate of 30 Mphotons pixel^−1^ s^−1^ at 12 keV with a linear response. Such a high count rate is achieved by a combination of a frame rate of 17.4 kHz and a peak signal of 1840 photons per pixel per frame at 12 keV. In an extended mode, where the exposure time is changed in alternating frames, CITIUS is designed to record 600 Mphotons pixel^−1^ s^−1^ at 12 keV. Single-photon detection capability is enabled by the system noise of 0.018 photons (60 e^−^ r.m.s.). Since the upgrade, the beamlines at the Extremely Brilliant Source of the European Synchrotron (ESRF-EBS) have pioneered the operation of fourth-generation synchrotrons, making them an excellent test bench for detector evaluation.

X-ray imaging detectors play a key role in the development of new measurement techniques that are required for full exploitation of the upgraded capabilities of fourth-generation synchrotron facilities. With the continuous rapid development of coherent X-ray sources and high-speed detectors, coherent diffraction imaging (CDI) methods are predicted to have a wide impact across a range of disciplines (Miao *et al.*, 2015 ▸). CDI is a high-resolution imaging method. An image is reconstructed from diffraction intensity without the use of downstream lenses, subject to coherence and oversampling conditions (Marchesini *et al.*, 2003 ▸). Under Bragg conditions, the technique can reveal the electron density and strain inside a crystalline sub-micrometre particle after applying phase-retrieval algorithms to the diffraction pattern collected around a Bragg peak of the particle. Fourth-generation synchrotrons such as ESRF-EBS produce brilliant and highly coherent X-rays so the technique is now detector limited. Detectors with a larger size and a greater dynamic range are needed to improve the spatial resolution of the technique, which is typically in the range of 5–10 nm or greater. Achieving a resolution near 1 nm and below is a critical issue in X-ray CDI for applications in materials. The practical resolution of hard X-ray CDI has mainly been limited by the intensity of the diffracted X-ray signal. The resolution was bound by the range of *q* space [*q* = (4π/λ)sinθ, where θ is half the scattering angle and λ is the wavelength of the incident radiation] within which the diffraction signal could be unambiguously determined from photon-counting noise. With ESRF-EBS, the resolution is now limited by the detector size and dynamic range. Such improvements provide scope for extending CDI to weaker X-ray events such as magnetic diffraction (Grimes *et al.*, 2022 ▸).

Here, we demonstrate the application of the high-speed charge-integrating detector CITIUS to Bragg CDI (BCDI). We compare BCDI results obtained with both a CITIUS detector and a Maxipix photon-counting pixel detector (Ponchut *et al.*, 2011 ▸). We then show how the CITIUS detector can be expected to perform in dynamic BCDI measurements and the performance of phase retrieval when the acquisition time is reduced by a factor of 1000. Finally, we discuss the current limitations of the CITIUS detector and further optimization for the BCDI technique.

## 2. Characteristics of the CITIUS detector

The CITIUS detector differs from the Maxipix in a few key regards. The CITIUS sensor possesses larger pixels (72.6 µm versus 55 µm) with a longer aspect ratio (728 × 324 pixels versus 516 × 516 pixels). The CITIUS detector operates at 17.4 kHz with a frame exposure time of 57.6 µs, while the Maxipix is limited to a frame rate of 0.1 kHz. Further details about the planned range of CITIUS detectors may be found in the technical literature (Hatsui *et al.*, 2023 ▸; Nishino *et al.*, 2023 ▸). On-the-fly conversion from deposited charge to instantaneous intensity was not fully implemented at the time of testing, being performed manually. This is addressed later in the *Discussion* section[Sec sec3].

## 3. Results and discussion

**Figure 1:**
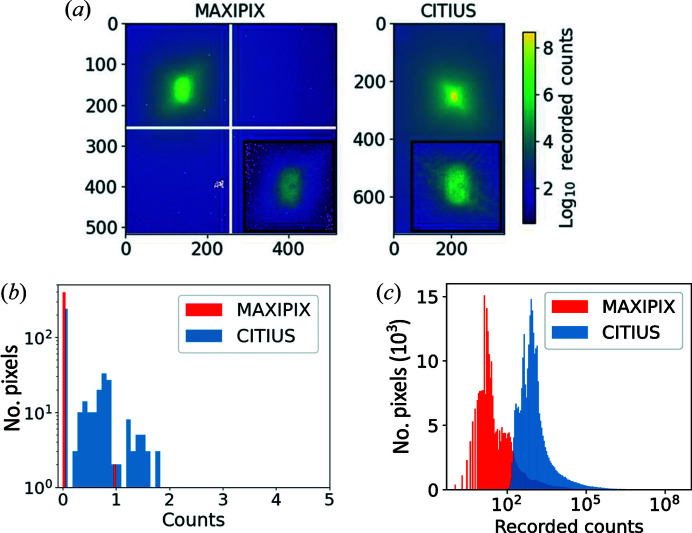
(*a*) A comparison between the recorded intensities of the Maxipix (1 s exposure) and CITIUS (0.1152 s exposure) detectors when a Siemens star sample is exposed to the direct beam of the ID01 source. The images shown are the sum total of the recorded counts across 401 frames of a ptychography scan. White pixels are located at the gaps of the Maxipix detector. A small portion of the upper left quadrant of the CITIUS sensor is masked, where hot pixels were seen to occur. Inset for each detector is a single frame of the ptychography scan. Histograms are provided (*b*) for the noise, taken for one frame over 20 × 20 pixels far from the illuminated area, and (*c*) for the observed counts per pixel over the full detector.

The CITIUS detector was tested under two experimental geometries on the ID01-EBS beamline at the ESRF at an X-ray energy of 8.5 keV (Leake *et al.*, 2019 ▸). At this photon energy, the noise of 60 e^−^ r.m.s. in the CCD data is equivalent to 0.026 photons r.m.s.; see Fig 1 ▸. In this manner, the performance of the CITIUS detector was evaluated (i) in transmission mode for ptychography and (ii) under Bragg conditions for BCDI. The resulting images are compared with those from a photon-counting detector (Maxipix), where frames are accumulated at a frequency of 33 Hz and attenuators are used to limit the maximum count rate per pixel to below 100 kcps to ensure linearity in the detector response.

### 3.1. Direct beam

Ptychography was performed by recording the direct beam following transmission (Hoppe, 1969 ▸; Pfeiffer, 2018 ▸) through a Siemens star sample which was incrementally translated in the *x* and *z* directions (perpendicular to the beam direction). The coherent beam was focused down using Be compound refractive lenses (CRLs). The focused beam size had FWHMs of 100 nm (horizontally) and 150 nm (vertically). The detectors were consecutively positioned on the detector arm at a distance of 1.28 m from the centre of the diffractometer. Al foil provided X-ray beam attenuation, with the thickness adjusted in each instance to prevent detector saturation. The dynamic ranges of the CITIUS (728 × 384 pixels of 72.6 µm, 0.1152 s exposure) and Maxipix (516 × 516 pixels of 55 µm, 1 s exposure) silicon detectors were compared by summing across 401 frames in each instance. The resulting images are shown in Fig. 1 ▸(*a*). Since the CITIUS is a charge-integrating detector, the number of X-ray photons was obtained by converting the collected charge into the number of X-ray photons. This calibration was done with single-photon events of known energy (Hatsui *et al.*, 2023 ▸). The maximum instantaneous intensities recorded at a single pixel were 38.01 Mcps pixel^−1^ for the CITIUS and 0.14 Mcps pixel^−1^ for the Maxipix. Averaging across the range of images results in an increase in the maximum photon flux by a factor of 158 for the CITIUS detector. This is attributed to the high dynamic range of the charge-counting detector. After correcting for the increased pixel size and detector thickness (Henke *et al.*, 1993 ▸), the instantaneous intensity remains at least two orders of magnitude greater. Note that Al foils were placed in the beam path to attenuate the X-ray intensity for the data acquired with the Maxipix detector to prevent saturation of detector pixels. Fig. 1 ▸(*b*) displays histograms of the noise taken from one frame over 20 × 20 pixels far from the illuminated area for the CITIUS and Maxipix detectors. The histograms demonstrate the differences in how detector counts are recorded: discrete versus continuous for the Maxipix and CITIUS, respectively. Fig. 1 ▸(*c*) shows log-plot histograms of recorded X-ray photon flux for each detector. The increased equivalent count rate is consistent across the entire data range with a shift in the peak of the histogram. The increase in the mean of the histogram (by a factor of 90) is almost completely compensated by the increase in the standard deviation of this value (by a factor of 90.5). This would suggest that the increase in X-ray exposure is not associated with any loss of contrast.

Comparable studies have previously been carried out where CDI experiments were performed using mixed-mode pixel array detectors, and the authors demonstrated how hybrid detectors can be used at 1 kHz frame rates (Tate *et al.*, 2013 ▸). The maximum count rate reported for ptychographic data in that paper was an order of magnitude lower than that of the CITIUS, demonstrating that charge-counting detectors are best placed to exploit the high brilliance of fourth-generation synchrotrons. Further scope for improvement lies in the reduction, and ideally removal, of detector gaps [see the central cross in Fig. 1 ▸(*a*)] where multiple sensors are used to increase the surface area of the detector. This can be achieved using shingled module construction, as recently implemented in the Pimega 540D detector (PITEC, https://www.pitec.co/wp-content/uploads/2021/03/20210302_PIMEGA_Brochure.pdf). In the medium- and far-field regimes, this effectively results in a gapless configuration. This is especially advantageous for techniques which require the signal to be recorded across a large range of 2D *q* space. One such example is BCDI, which is discussed further in the next section.

### 3.2. Application to Bragg CDI

The sample consists of Pt nanoparticles grown on an yttria-stabilized zirconium (YSZ) substrate. The three-dimensional Bragg coherent diffraction imaging (BCDI) measurement was performed by collecting the scattered intensity in the vicinity of the specular 111 Pt Bragg reflection. The sample was mounted on a fast *xyz* piezoelectric stage with a lateral stroke of 100 µm and a resolution of 2 nm, sitting on a hexapod that was mounted on a (3 + 2 circle) goniometer. The beam size was focused down using CRLs. The resulting beam size was approximately 1 µm (horizontally) × 1 µm (vertically). The scattered X-rays were detected using first a Maxipix detector and then a CITIUS detector. The detectors were positioned consecutively on the detector arm at a distance of 0.7 m from the sample. We measured the 111 Pt Bragg reflection in three dimensions by rotating a Pt particle around the Bragg angle through 1.6° in 200 steps of 0.008°. The counting time was 1 s for each step of the rocking curve for the Maxipix detector. Two thousand frames of 57.6 µs (running at 17.4 kHz) at each step of the rocking curve were acquired for the CITIUS detector. This implies that the acquisition time was 0.1152 s for each step of the rocking curve, *i.e.* 8.7 times shorter than that used with the Maxipix.

**Figure 2:**
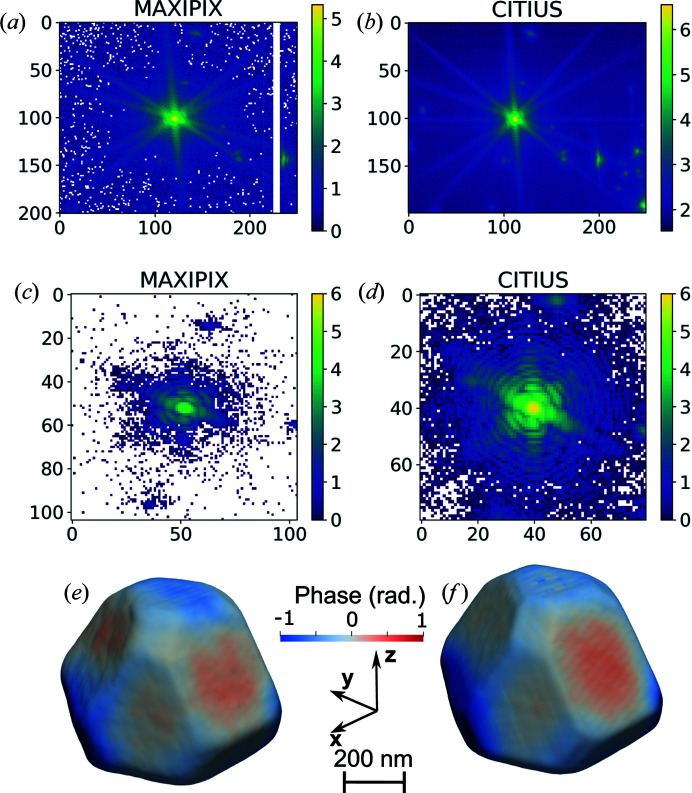
(*a*), (*b*) Comparisons between the recorded intensities on a logarithmic scale on (*a*) the Maxipix detector (1 s per step) and (*b*) the CITIUS detector (0.1152 s per step) for the 111 Bragg peak scattered from the same Pt particle. White pixels indicate the detector gap or zero-value pixels. The 3D signal has been summed across the out-of-plane rocking angle. (*c*), (*d*) Slices at the maximum of the rocking curve shown on a logarithmic scale for (*c*) the Maxipix and (*d*) the CITIUS detector. The shown slices from each detector have equivalent surface area, where the difference in pixel size is taken into account. (*e*), (*f*) Three-dimensional views of the isosurface of the reconstructed phase of the Pt particle, where the boundary is drawn at 50% of the maximum density, using data collected with (*e*) the Maxipix and (*f*) the CITIUS detector.

Figs. 2 ▸(*a*) and 2 ▸(*b*) display CDI measurements of the 111 Pt Bragg peak for the same Pt particle summed along one direction of the detector acquired with the Maxipix and CITIUS detectors. Al foil was placed in the beam path to attenuate the X-ray intensity for the data acquired with the Maxipix detector to prevent saturation of detector pixels. Conversely, data recorded with the CITIUS detector had no such beam attenuation. This resulted in a higher instantaneous intensity measured with the CITIUS detector (8.2 Mcps pixel^−1^) than with the Maxipix detector (0.04 Mcps pixel^−1^). The same streaks originating from the faceted Pt crystal are captured by the Maxipix and CITIUS detectors; see Figs. 2 ▸(*a*) and 2 ▸(*b*), respectively. Figs. 2 ▸(*c*) and 2 ▸(*d*) show slices at the maximum intensity of the 111 Pt Bragg peak collected with the Maxipix and CITIUS detectors, respectively. These images indicate the relative maximum intensities in each detector for BCDI measurements. Notably, the diffraction signal on the CITIUS detector [Fig. 2 ▸(*d*)] can be distinguished over a larger surface area, where the acquisition time was 11% that of the Maxipix detector [Fig. 2 ▸(*c*)]. Furthermore, attenuation was not necessary for the CITIUS detector.

Phase-retrieval algorithms were applied to retrieve the Bragg electron density (shape) and phase within the single Pt nanoparticle through the use of the *PyNX* package (Favre-Nicolin *et al.*, 2011 ▸, 2020*a* ▸). Phase retrieval was carried out on the raw diffracted intensity data. The initial support, which is the constraint in direct space, was first estimated from the auto-correlation of the diffraction intensity. The following minimization algorithms were implemented using this initial support: 1000 relaxed averaged alternating reflection (RAAR; Luke, 2004 ▸) steps, 400 hybrid input output (HIO; Fienup, 1978 ▸) steps and 300 error-reduction (ER; Gerchberg & Saxton, 1972 ▸; Fienup, 1978 ▸) steps, while a shrink wrap algorithm was iteratively applied (Marchesini *et al.*, 2003 ▸). The phasing process included a partial coherence algorithm to account for the partially incoherent incoming wavefront (Clark *et al.*, 2012 ▸). Following phase retrieval, the ten best reconstructions (those with the lowest free log-likelihood; Favre-Nicolin *et al.*, 2020*b* ▸) were selected from 50 runs using random phase starts. Next, five reconstructions were further selected from the ten where the criterion was the lowest value of the standard deviation of the electron density. Finally, we performed the decomposition into modes from these last five reconstructions (Favre-Nicolin *et al.*, 2020*b* ▸). Final corrections to account for refraction and absorption were performed using the *bcdi* package (Carnis *et al.*, 2022 ▸). After removal of the phase ramp and phase offset, the data were finally interpolated onto an orthogonal grid for ease of visualization.

Figs. 2 ▸(*e*) and 2 ▸(*f*) display 3D views of the isosurface of the reconstructed phase of the Pt particle drawn at 50% of the maximum density from retrieved data collected with the Maxipix and CITIUS detectors, respectively. The particle has a size of 650 × 650 × 400 nm. The 3D phases retrieved from the data collected from the CITIUS and Maxipix detectors are in good agreement, even though the acquisition time for the CITIUS data was 8.7 times shorter than that used with the Maxipix, for which Al foils were used so as not to saturate the detector.

**Figure 3:**
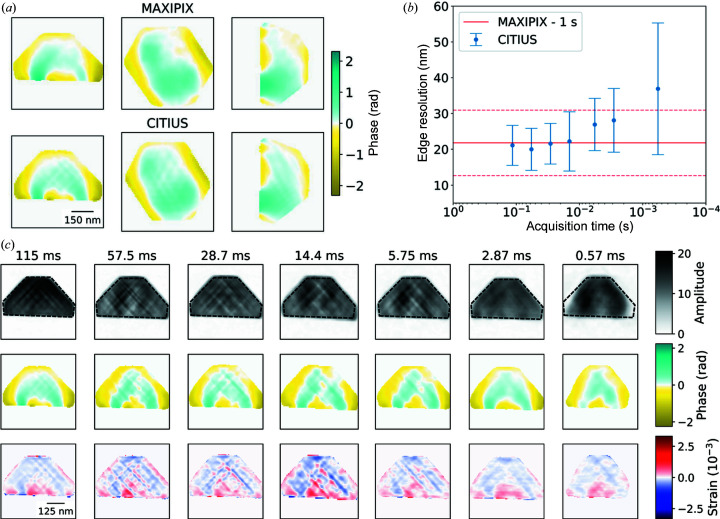
BCDI phase retrieval. (*a*) A comparison between reconstructed object phases using data acquired with (top) the Maxipix detector (1 s exposure) and (bottom) the CITIUS detector (0.1152 s exposure). (*b*) The quality of phase retrieval is compared by quantifying the edge resolution of the reconstructed nanoparticles. Time refers to the acquisition time of each point in the rocking curve. Resolution is estimated as the width of the first derivative of the particle amplitude at each edge, being averaged across the *x*, *y* and *z* directions. (*c*) Slices across the *y* axis of the reconstructed objects. The amplitude, phase and out-of-plane strain of the Pt particles are reconstructed using reduced data frames (from the CITIUS detector) to simulate reduced acquisition times. The quality of the reconstructions remains high above 5 ms per point.

A direct comparison between the object phase reconstructed from the Maxipix and CITIUS data is presented in Fig. 3 ▸(*a*). The phases of the object displacement are nearly identical between the two detectors. In order to compare the quality of the reconstructed objects, one has to consider both the voxel size of the object and the sharpness of the features. For the purposes of comparison, we compute the sharpness of the object facets in each direction. This is implemented by fitting a Gaussian function to the first derivative of the object amplitude with respect to the object axes. The means of these boundary sharpnesses are reported as the edge resolutions, which can be compared irrespective of voxel size. Under the experimental conditions outlined above, this results in an edge resolution of 20 ± 6 nm for the object reconstructed from data obtained using the CITIUS detector, compared with 22 ± 9 nm for the data obtained with the Maxipix detector.

As a consequence of the recording procedure, we can simulate reduced acquisition times by using fewer frames from the raw data, *i.e.* 100 frames ≃ 5.75 ms acquisition time. In this manner, we can demonstrate how the CITIUS detector could be expected to perform in dynamic measurements far beyond the capability of photon-counting detectors (typically 1 s for the Maxipix detector). In a typical BCDI scan, 200 images (×1 s) are recorded to construct a 3D Bragg peak, resulting in a scan time of ∼3 min to generate an image of a 650 nm diameter Pt nanoparticle with 20 nm resolution. The edge resolutions of objects with simulated reduced acquisition time using a CITIUS detector are shown in Fig. 3 ▸(*b*). The solid line corresponds to the resolution of the Maxipix data with a standard count time of 1 s (1 frame s^−1^). This demonstrates how the quality of the CITIUS data degrades with reduced acquisition times. However, the reduction in quality is only visible below 0.01 s. Indeed, the quality is only significantly reduced below 1 ms of acquisition time per point. In Fig. 3 ▸(*c*) we show the reconstructed objects when varying counting times. The general features of the particle (shape, size *etc.*) are still discernible with an acquisition time of 0.57 ms per image, albeit with significantly reduced spatial resolution. This demonstrates how the next generation of detectors can improve the dynamic range of the BCDI technique, where phase retrieval could be performed with total scan times of 0.11 s (0.57 ms × 200 points for a rocking curve). This would allow users to take full advantage of the increased capabilities of fourth-generation synchrotrons.

This work demonstrates the possibility of probing particle structure on the millisecond time scale without drastically degrading the quality of the reconstructed data (for the shown example of a 650 × 650 × 400 nm Pt particle). Previously, the X-ray dose at synchrotrons has been reduced using photon-sparse random projections, with illumination times of 25 ms per point (Giewekemeyer *et al.*, 2019 ▸). The CITIUS detector offers a significant reduction in acquisition times without loss of spatial resolution.

Further optimization of the CITIUS detector is ongoing to advance lensless imaging performed at fourth-generation synchrotrons, including (i) a larger detector size with minimal gaps to improve the spatial resolution of the BCDI technique (the spatial resolution being inversely proportional to the measured volume in reciprocal space) and (ii) the ability to work in burst mode with effectively no dead time (*i.e.* a fully integrated detector) as BCDI demands the acquisition of the entire 2D image. Currently, the optical fibres from the proximity electronics near the CITIUS sensor to three PCIe boards, namely data-framing boards (DFBs), inside the server have a large enough bandwidth to transmit all the raw data. The bottleneck is between the DFBs and the server, where the data are transmitted via a total of three PCIe Gen3 x8 slots. One way to circumvent the bottleneck is to implement an additional PCIe Gen3 x8 cable for each DFB, which has been done to demonstrate the performance where all the data are transmitted to the server memory. Another more promising approach is to conduct on-the-fly data analysis by the field-programmable gate array (FPGA) on the DFB. Detailed implementation of the data acquisition scheme and its performance are to be reported elsewhere (Nishino *et al.*, 2023 ▸).

## 4. Conclusions

In summary, we have demonstrated the application of the CITIUS detector for Bragg coherent diffraction imaging. The performance of this new generation of charge-integrating detector is compared with that of a photon-counting device. The same quality is observed for data acquired with the Maxipix and CITIUS detectors, the CITIUS detector having the advantage of a higher dynamic range and a higher frame rate.

Charge-integrating detectors open the door to imaging a wide range of dynamic phenomena with high spatio-temporal resolution.
